# Supplementary material for: Surfactin and Spo0A-Dependent Antagonism by Bacillus subtilis Strain UD1022 against Medicago sativa Phytopathogens
Source: Plants (Basel). 2023 Feb 23;12(5):1007. doi: 10.3390/plants12051007 (PMC10005099; doi:10.3390/plants12051007)
Supplement: Supplementary file 1 [file plants-12-01007-s001.zip › plants-2222730-supplementary.pdf]

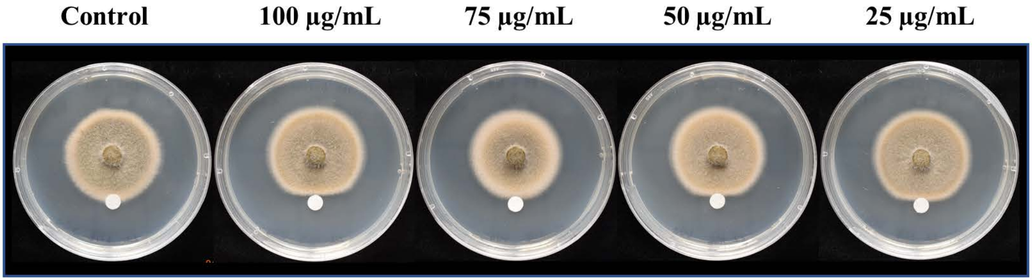

Supplementary Figure S1. Surfactin disk challenge of A. medicaginicola StC 306-5. Surfactin mildly but not significantly antagonized StC 306-5 colonies at 25 and 100 g/ml.

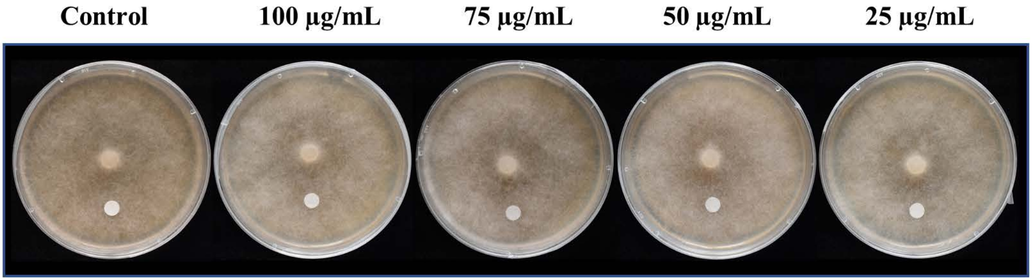

Supplementary Figure S2. Surfactin disk challenge of Phythophthora medicaginis A2A1. Direct application of surfactin had no effect on A2A1 growth at 25 g/ml to 100 g/ml.

Supplementary Table S1. B. subtilis UD1022 Mutants. Bacterial strains used in this study.

Strain	Genotype
UD1022 spo0A	spo0A::erm
UD1022 sinI	sinI::spc
1022 eps tasA	eps::tet<br>tasA::erm
UD1022 srfAC	srfAC::erm
UD1022 sfp	sfp::erm
UD1022 ppsB	Ω tn10::spec
UD1022 ppsB srfAC	Ω tn10::spec<br>srfAC::erm
